# Expressed breast milk and maternal expression of breast milk for the prevention and treatment of neonatal hypoglycemia: a systematic review and meta-analysis

**DOI:** 10.1186/s40748-023-00166-0

**Published:** 2023-10-09

**Authors:** Oluwatoyin Ibukun Oladimeji, Jane E Harding, Caroline A Crowther, Luling Lin

**Affiliations:** Liggins Institute, The University of Auckland, Auckland, New Zealand

**Keywords:** Expressed breast milk, Breast milk expression, Neonate, Hypoglycemia, Prevention, Treatment

## Abstract

**Background:**

Worldwide, many guidelines recommend the use of expressed breast milk (EBM) and maternal expression of breast milk for the prevention and treatment of neonatal hypoglycemia. However, the impact of both practices on neonatal hypoglycemia is unclear. This study aims to determine the effectiveness of EBM and maternal expression of breast milk in preventing and treating neonatal hypoglycemia.

**Methods:**

We registered our review in PROSPERO (CRD42022328072). We systematically reviewed five databases and four clinical trial registries to identify randomized controlled trials (RCT), non-randomized studies of intervention (NRSI), and cohort studies that compared infants who received EBM to infants who did not, and similar study designs that compared infants whose mothers expressed breast milk to infants whose mothers did not. Two independent reviewers carried out screening, data extraction, and quality assessment. The quality of included RCT, NRSI, and cohort studies were respectively assessed with the Cochrane Risk of Bias 2, Risk Of Bias In Non-randomised Studies—of Interventions, and the Newcastle–Ottawa Scale tools. Results from studies on EBM were synthesized separately from those on maternal expression of breast milk. Meta-analysis was undertaken using Revman 5.4. and fixed-effect models.

**Results:**

None of the ten included studies was specifically designed to determine the effect of EBM or maternal expression of breast milk on neonatal hypoglycemia. The effect of EBM on neonatal hypoglycemia was not estimable. There was no difference in the risk of hypoglycaemia among neonates whose mothers expressed breast milk compared to those whose mothers did not [RR (95%CI); one RCT: 0.92 (0.77, 1.10), high-certainty evidence; one cohort: 1.10 (0.74, 1.39), poor quality study].

**Conclusions:**

There is insufficient evidence to determine the effectiveness of EBM for preventing or treating neonatal hypoglycemia. Limited data suggests maternal breast milk expression may not alter the risk of neonatal hypoglycemia. High-quality randomized controlled trials are needed to determine the effectiveness of EBM and maternal expression of breast milk for the prevention and treatment of neonatal hypoglycemia.

**Supplementary Information:**

The online version contains supplementary material available at 10.1186/s40748-023-00166-0.

## Background

Neonatal hypoglycemia is the most common metabolic disorder among newborn infants, affecting 5% to 15% of these infants [1] and approximately one in two of all at-risk infants [2]. The risk of neonatal hypoglycemia is highest in the first few hours after birth. This is because clamping of the umbilical cord at birth leads to termination of transplacental glucose transfer coupled with continued endogenous production of insulin by the infant [3, 4]. The risk of hypoglycemia is increased in states associated with reduced glycogen stores, increased glucose utilization, and hyperinsulinemia [3, 5]. In these states, compensatory mechanisms like the production of counterregulatory hormones (cortisol and glucagon), which trigger gluconeogenesis and glycogenolysis thus resulting in endogenous glucose production, are often delayed [3]. Risk factors associated with such states include being small or large for gestational age, an infant of a diabetic mother, preterm, asphyxiated, and hypothermic [3, 5]. Hence, a prompt exogenous supply of glucose may be important in preventing neonatal hypoglycemia, especially in at-risk infants. Despite being the commonest metabolic disorder in newborn infants, there is no consensus on the threshold that define neonatal hypoglycaemia [3, 6]. However, its most widely accepted definition is blood glucose concentration less than 47 mg/dL (2.6 mmol/L), with variations even among pediatric professional organizations [6].

Hypoglycemia in the newborn, especially when severe, recurrent, or not promptly detected and treated, is associated with far-reaching poor perinatal and long-term neurodevelopmental outcomes [7, 8]. These include neonatal seizures, apnea, death, developmental delays, seizure disorder, visual-motor impairment, and executive dysfunction [5, 7–9]. Prevention and management options for neonatal hypoglycemia include breastfeeding [10], oral glucose gel [11], intravenous dextrose [4], medications such as hydrocortisone and glucagon [4], and feeding with formula milk or expressed breast milk (EBM) [10].

Both feeding EBM (mother’s or donor’s) to infants and the expression of breast milk by mothers to prevent or treat hypoglycemia are incorporated into many neonatal management guidelines worldwide [5, 10, 12–14]. While EBM provides ready non-formula feeds for the infant, the expression of breast milk, in addition, may be associated with improved lactogenesis [15]. Thus, these two interventions, although closely related, may potentially have independent effects on neonatal hypoglycemia. Both practices are recommended, increasingly encouraged, and practised for at-risk and hypoglycemic infants [10, 12–14, 16], yet their effectiveness in preventing and treating neonatal hypoglycemia is uncertain. Hence, this systematic review aims to review the evidence on the effectiveness of feeding EBM and maternal expression of breast milk for preventing and managing neonatal hypoglycemia.

## Methods

We registered our study protocol in the International prospective register of systematic reviews (PROSPERO)-CRD42022328072 [17]. In addition to investigating the effectiveness of EBM for the prevention and treatment of neonatal hypoglycemia, our registered protocol was revised (expanded) to determine the effectiveness of maternal expression of breastmilk for the prevention and treatment of neonatal hypoglycemia as this is also a commonly recommended practice in neonatal care [12, 14]. Hence, our protocol was expanded to include relevant review questions, participants, interventions, and comparators [17]. Our review is reported following the Preferred Reporting Items for Systematic Reviews and Meta‐Analyses (PRISMA) guidelines [18].

### Eligibility criteria

We included studies that compared infants (≤ 28 days old) who received EBM (mother’s or donor’s) to infants who received no intervention or other interventions (breastfeeding, formula milk, dextrose gel, intravenous dextrose, placebo, or a combination of these) as well as studies that compared infants of mothers who expressed breast milk with infants whose mothers did not express breast milk. Randomized controlled trials (RCTs), quasi-RCTs, non-randomized studies of intervention (NRSI), cluster randomized trials, cohort and case–control studies, and abstracts (if they provided enough information) were included. There were no language or geographic restrictions. Study protocols and those without comparison groups were excluded.

### Search strategy

We searched OVID MEDLINE, Embase (OVID), CINAHL Plus, Cochrane Library, and Scopus from inception to 19^th^ May 2022, and trial registration repositories, Current Controlled Trials, Clinical Trials, Australian and New Zealand Clinical Trials Registry, and the World Health Organization International Clinical Trials Registry Portal (Additional file 1). In addition, we searched references of previous relevant reviews for additional studies for relevant articles. Results from the literature search were imported into Covidence software [19], where studies were screened. Two authors (OIO and JH/LL) independently reviewed all studies for eligibility. Any discrepancies were resolved after discussion or involving a third author (JH/LL).

### Study selection

We included all RCTs, NRSI, and cohort studies that compared infants given EBM to those given no or other interventions and studies that compared infants whose mothers expressed breast milk with those whose mothers did not express breast milk. We did not identify any relevant case–control study.

The primary outcome was neonatal hypoglycemia (study-defined, i.e., as defined by study authors) after the intervention. Secondary outcomes were neonatal hypoglycemia (any blood glucose concentration ≤ 2.6 mmol/L), receipt of treatment for hypoglycemia (study-defined), number of episodes of hypoglycemia (study-defined), severity of hypoglycemia (lowest recorded blood glucose concentration or study-defined), separation from the mother for any treatment before discharge home (infant nursed in an environment not in the same room as the mother, e.g., for neonatal intensive care unit (NICU) admission or special care baby unit (SCBU) admission), separation from the mother for treatment of hypoglycemia before discharge home (infant nursed in an environment not in the same room as the mother, e.g., NICU admission or SCBU for treatment of hypoglycemia), injury attributable to hypoglycemia on brain imaging (study defined), duration of initial hospital stay, breast milk feeding exclusively (infant only receives breast milk without any other drink or food) from birth to discharge, breast milk feeding exclusively after discharge, breastfeeding (any) after discharge, exclusive breast milk feeding (infant only receives breast milk without any other drink or food) at six months after birth, cost of intervention (as measured by study), cost of neonatal care (as measured by the study).

### Data extraction, synthesis, and analysis

Two authors (OIO and LL) independently extracted data using pre-designed data extraction forms. Data extracted include study design, location, year of publication, population, intervention used, control exposure, and whether the study was primarily designed to prevent or treat hypoglycemia. The risk of bias for outcomes was independently assessed by two authors (OIO and LL) using the Cochrane Risk of Bias -2 tool [20] for RCTs, Risk Of Bias In Non-randomized Studies of Interventions tool (ROBINS-I) [21] for NRSI, and the Newcastle–Ottawa Scale [22] for cohort studies. For RCTs and NRSI, the risk of bias was assessed for each outcome, while for cohort studies, the risk of bias was assessed for each study. For RCTs, study outcomes were assessed as having low, some concerns or high risk of bias [20], while for NRSI, they were assessed as having low, moderate, serious, or critical risk of bias [21]. Cohort studies were assessed as being of good or poor quality [22]. Discrepancies were resolved with discussion. We planned to assess publication bias by visual inspection of a funnel plot, plotting the study effect size against the sample size, but this was not possible because of few relevant studies.

We calculated the risk ratio (RR) with 95% confidence intervals (CIs) for dichotomous outcomes and the mean difference (MD) with 95% CIs were calculated for continuous outcomes. A *p*-value of < 0.05 denoted statistical significance. The median (first and third quartiles) were converted to mean (SD) for studies that report median [23]. The mean (SD) for studies with two or more EBM groups (e.g. raw vs. pasteurized) were merged to create a single group as recommended by the Cochrane Collaboration [24]. For studies that presented results using graphs, WebPlotDigitizer was used to extract numbers from the graph [25].

Meta-analysis is a valid, accurate and precise method for synthesizing estimates reported by at least two studies [26, 27]. Hence, for outcomes reported by a minimum of two studies, meta-analysis was undertaken using Revman 5.4 [28] and fixed-effect models. I^2^ and χ^2^ were calculated for each analysis and describe the percentage of variability in effect estimates due to heterogeneity. If we observed substantial heterogeneity (I^2^ > 50% and *P* < 0.10 in the χ^2^ test), we planned to explore possible causes in a sensitivity analysis.

Grading of Recommendations Assessment, Development and Evaluation (GRADE) [29] was used to assess the certainty of evidence for RCTs reporting any of the following outcomes: neonatal hypoglycemia (study-defined), receipt of treatment for hypoglycemia (study‐defined, any treatment ‐ oral dextrose gel, intravenous dextrose, or other drug therapy) during the initial hospital stay, separation from the mother for any treatment before discharge home, separation from the mother for treatment of hypoglycemia before discharge home, breast milk feeding exclusively from birth to discharge, exclusive breast milk feeding at six months. Results from studies on EBM were synthesized and reported separately from those on expression of breast milk.

## Results

**Fig. 1:**
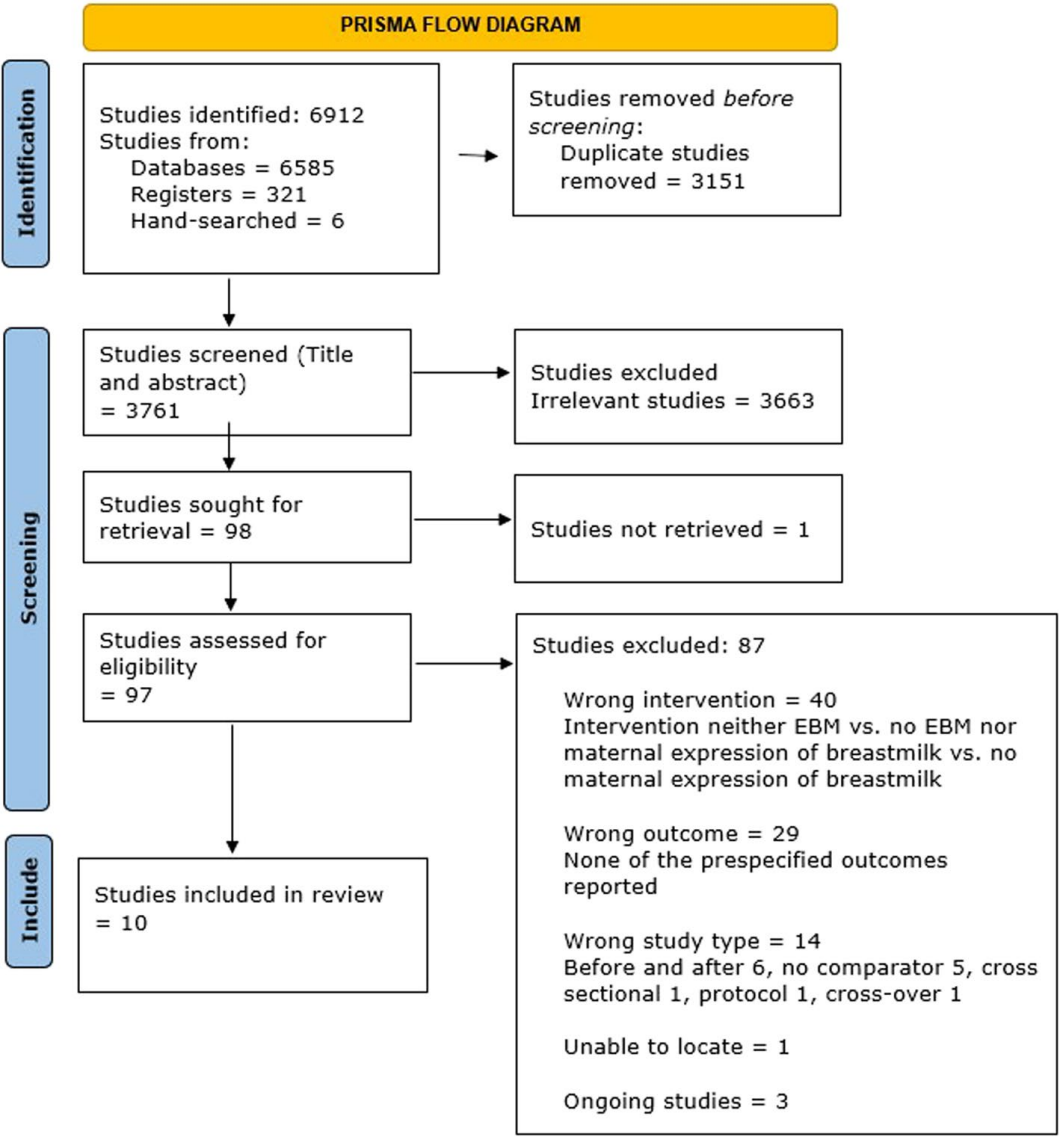
Flow diagram of the process of study selection

We identified 6 912 studies, of which six were additional papers identified through a review of references (Fig. 1). After removing duplicates, 3 761 studies were screened. After title and abstract screening, 3 663 studies were excluded. One study could not be retrieved despite contacting the authors. Of the remaining 97 studies for which we conducted full text review, we included 10 studies, two of which were included in the meta-analysis and the remaining eight were included in the qualitative analysis. None of the studies specifically investigated the use of EBM or breast milk expression for preventing and treating neonatal hypoglycemia. Of the three ongoing studies [30–32], one [30] is investigating the effectiveness of human donor milk for the treatment of neonatal hypoglycemia among breastfed infants.

### Study characteristics

The included studies were five RCTs [16, 33–36], two NRSIs [37, 38], and three cohort studies [39–41] (Table 1). Five of the studies were about the effects of EBM and five about maternal expression of breast milk. The majority (90%) were conducted in high-income countries, while one (10%) was conducted in a low-middle income country (India). The publications spanned more than six decades (1958 to 2022), with sample sizes ranging from 20 to 656 infants.

**Table 1:** Characteristics of included studies

**Study details** Author (year published) Duration Location	**Design**	**Population**	**Intervention/Exposure [N]**	**Control [N]**	**Outcome measure of interest**	**Study primarily designed for the prevention and or treatment of neonatal hypoglycemia**	**Authors’ conclusion**
**Studies on expressed breast milk**							
Cossey (2014) [37] September 2006 to March 2008 Leuven, Belgium	Non-randomized study of intervention	Infants born before 32 weeks of gestation and/or below 1 500 g	Expressed breast milk—mother’s either raw or pasteurized [106]	Pre-term formula [44]	Duration of initial hospital stay	No	Using different milk diets as tools to influence the colonization process does not modify the prevalence, density, or stability of the staphylococcal colonization
Cristofalo (2013) [33] July 2007 to July 2008 Seven NICUs (6 in the US and 1 in Austria)	Multi-centre randomized controlled trial	Extremely preterm infants born at 25 to 29 weeks whose mothers did not provide their milk	Expressed breast milk—pasteurized donor milk, appropriately fortified human milk [29]	Bovine milk–based preterm formula [24]	Duration of initial hospital stay	No	This trial supports the use of an exclusive human milk diet to nourish extremely preterm infants in the neonatal intensive care unit
Harris (2017)[39] December 1, 2008, to November 26, 2010 A tertiary referral centre (Waikato Women’s Hospital) in Hamilton, New Zealand	Cohort	Infants born at 35 -42 weeks and ≤ 48 h old who had hypoglycemia	Expressed breast milk (mother’s) after prior receipt of either dextrose gel or placebo [105]	Breastfeeding or formula or no milk after prior receipt of either dextrose gel or placebo [122]	Change in blood glucose concentration after different oral treatments for hypoglycemia	No	Treatment with dextrose gel 200 mg/kg or infant formula, but not expressed breast milk or breast feeding alone, are associated with a significantly greater increase in blood glucose concentration than would occur without treatment in infants with hypoglycemia in the first 48 h after birth
Narayanan (1981) [34] Not stated A neonatal special care unit in New Dehli, India	Randomized controlled trial	Premature low birth-weight infants at risk of neonatal infections	Expressed human milk- mother’s or donor’s [31]	Nursery formula (LactodexRaptakos and Brett) [31]	Duration of initial hospital stay	No	Expressed human milk is particularly useful for infants who are at high risk for infection
Schultz (1980) [35] Not stated Premature ward, Hungary	Randomized controlled trial	Low birth-weight infants born at 30 to 37 weeks	Pooled mature human milk [10]	Cow milk protein based standard formula (Robolact) [10]	Neonatal hypoglycemia Fasting blood glucose concentrations	No	Human milk provides a safe nutritional management in the early postnatal life, although further research is needed of how human milk should be supplemented for preterm infants
**Studies on expression of breast milk**							
Casey (2019) [40] 2014 to 2015 North Queensland, Australia	Retrospective cohort	Pregnant women with diabetes (GDM and pre-existing diabetes) and their infants	Expression and storage of antenatal colostrum [80]	No expression and storage of antenatal colostrum [223]	Neonatal hypoglycemia Exclusive breastfeeding at discharge Duration of initial hospital stay	No	No independent association was found between antenatal expression of colostrum and the rates of neonatal hypoglycemia or median blood glucose levels. Expressing antenatal colostrum may have some benefits to the infant such as reduced formula consumption in hospital
Demirci (2022) [36] December 2016 to February 2018 A hospital-based midwife practice in the United States	Randomized controlled trial	Low-risk, nulliparous pregnant individuals	Antenatal expression of milk [18]	Lactation education without antenatal expression of milk [18]	Separation from the mother for any treatment before discharge home i.e., NICU admission Breastfeeding exclusively after discharge (1–2 weeks and 3–4 months)	No	Antenatal milk expression (AME) education and independent practice beginning at 37 weeks of pregnancy was feasible. In some cases, AME provided a back-up supply of milk when supplementation was indicated or desired. The relationship between AME and lactation outcomes requires further study with adequately powered samples
Forster (2017) [16] June 6, 2011, and Oct 29, 2015 Six hospitals in Victoria, Australia	Multicentre, two-group, unblinded, randomized controlled trial	Pregnant women with diabetes (pre-existing diabetes or gestational)	Antenatal expression of milk [317]	Standard pregnancy care and advice without antenatal expression of milk [315]	Neonatal hypoglycemia Separation from the mother for any treatment before discharge home i.e., NICU admission Separation from the mother for treatment of hypoglycemia before discharge home Exclusive breastfeeding from birth to discharge (or to 7 days if still inpatient at that time point) Breastfeeding exclusively after discharge (at 3 months)	No	There is no harm in advising women with diabetes in pregnancy at low risk of complications to express breast milk from 36 weeks’ gestation
Ingelmann-Sunderberg (1958) [38] 01/01/1951 to 01/06/1953 Stockholm, Sweden	Non-randomized study of intervention	Obstetric women at a private lying-in ward	Antenatal expression of colostrum [313]	Regular washing of breast with soap and water [343]	Exclusive breastfeeding at discharge	No	Antenatal massage of nipples and expression of colostrum is of no value as a routine treatment and should be used only in cases with poorly protractile and retrotractile nipples
Soltani (2012) [41] 2001–2003 Derby Hospitals NHS Foundation Trust, United Kingdom	Retrospective cohort	Pregnant women diabetes (type I, type II or gestational)	Antenatal expression of breast milk [16]	No antenatal expression of breast milk [69]	Separation from the mother for any treatment before discharge home i.e., SCBU admission	No	There seems to be a trend between antenatal breast milk expression and lower gestational age at birth. The trend of a higher rate of SCBU admission for infants from the breast milk expression group compared to those who did not express antenatally, is of concern

The number of infants included in this review was 2 224. The five studies on EBM included 512 infants, of whom 281 received EBM (mother’s [211], donor [29], mother’s or donor [31], or unspecified [10]) and 231 received other interventions (formula [109], or a combination of breastfeeding, no milk and formula [122]). The five studies on breast milk expression included 1 712 infants, 744 mothers of whom expressed breast milk, and 968 did not. All mothers who expressed breast milk did so antenatally. Three studies included mothers with pre-gestational or gestational diabetes [16, 40, 41], one involved low-risk nulliparous individuals [36] and one involved mothers in an obstetric ward [38].

### Risk of bias

The risk of bias by outcomes reported by RCTs and NRSI for studies on expressed breast milk and maternal expression of breast milk varied widely from low to high risk of bias (Table 2).

**Table 2:**
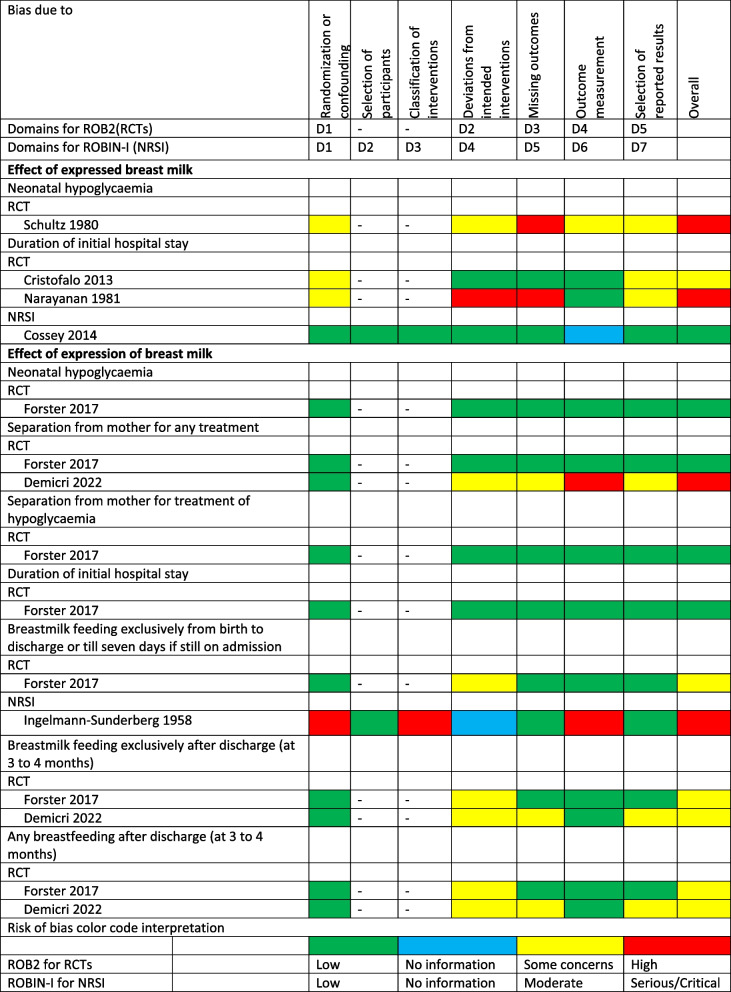
Risk of bias for outcomes reported in randomized controlled trials and non-randomized studies of interventions

*RCTs* Randomized Controlled Trials, *NRSI* Non-randomized Study of intervention, *ROB2* Cochrane risk of bias 2 tool,

*ROBINS-I* Risk Of Bias In Non-randomized Studies of Interventions tool, D – Domain, “- “not separately assessed

Similarly, the only cohort study that reported on EBM and a relevant outcome was of good quality, while the two cohort studies that reported on maternal expression of breast milk and relevant outcomes were of poor quality (Table 3).

**Table 3:**
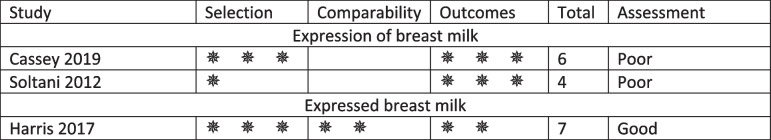
Risk of bias for cohort studies

Quality assessment of included studies. Good quality: 3 or 4 stars in selection domain AND 1 or 2 stars in compatibility domain AND 2 or 3 stars in outcome/exposure domain, Fair quality: 2 stars in selection domain AND 1 or 2 stars in comparability domain AND 2 or 3 stars in outcome/exposure domain, Poor quality: 0 or 1 star in selection domain OR 0 stars in comparability domain OR 0 or 1 stars in outcome/exposure domain

### Outcomes for studies on expressed breast milk

#### Neonatal hypoglycemia

**Fig. 2:**
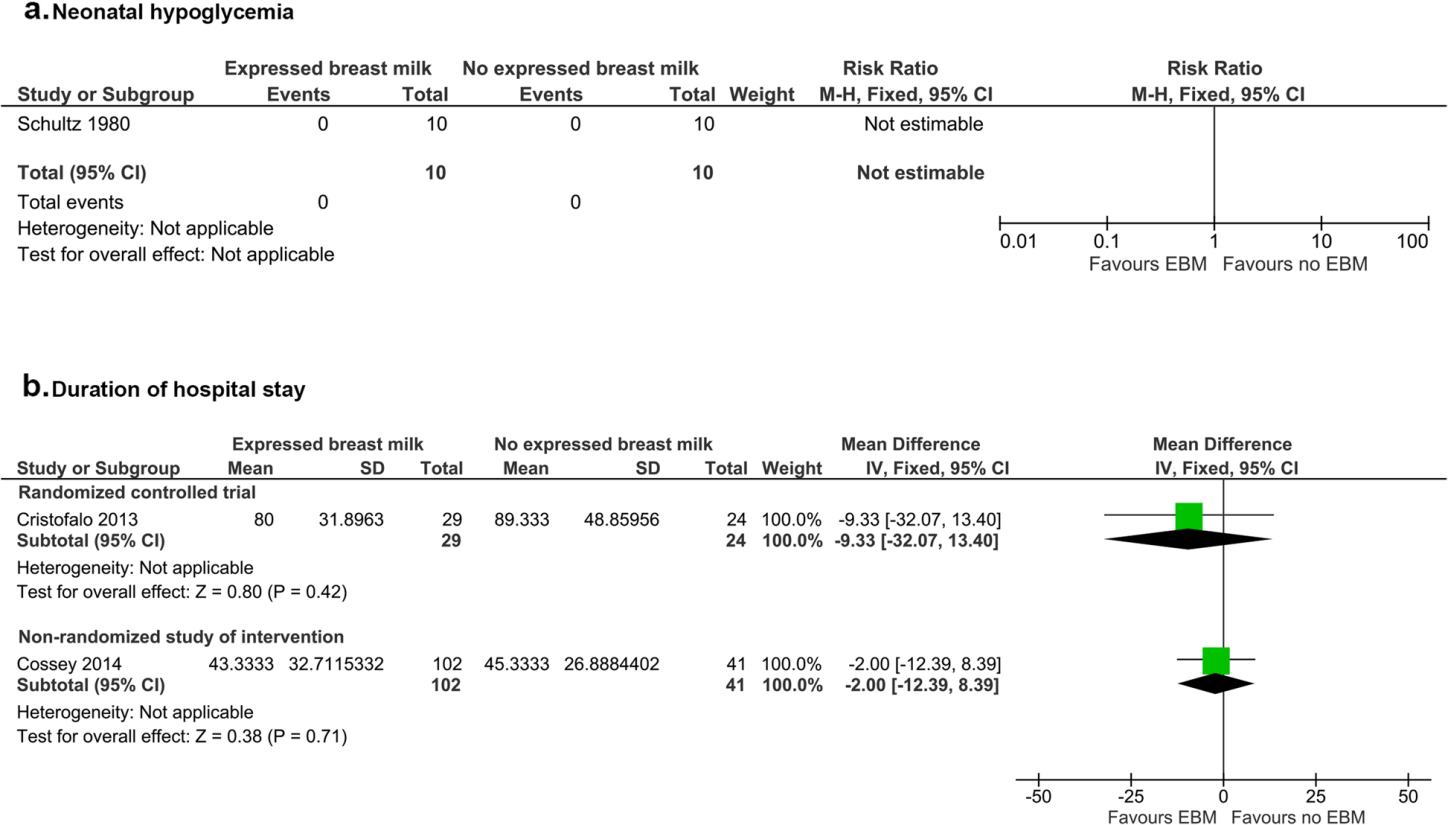
Expressed breast milk and relationship with neonatal hypoglycemia and duration of initial hospital stay

One RCT [35] reported no hypoglycemic episodes in infants who were and were not given EBM, although authors did not report any blood glucose concentrations nor explain how hypoglycemia was defined (20 infants, RR – not estimable, very low certainty evidence, high risk of bias) (Fig. 2).

#### Duration of initial hospital stay

Three studies [33, 34, 37] compared the duration of hospital admission among infants given EBM and infants given other interventions. One RCT [33] reported no difference in the duration of initial hospital stay of infants who were fed EBM compared to infants who had other interventions (53 infants, MD [95% CI]: -9.33 [-32.07, 13.40] days, *p* = 0.42, some concerns about risk of bias) (Fig. 2). Similarly, one NRSI [37] reported the duration of hospital stay among infants fed expressed mother’s milk was not different from infants fed infant formula (143 infants, MD [95% CI]: -2.00 [-12.39, 8.39] days, *p* = 0.71, low risk of bias). One RCT [34] reported that among infants who developed an infection, the duration of initial hospital stay was shorter among infants given breast milk compared to infants given formula, but no supporting data or statistical measures were reported (62 infants, MD not estimable, high risk of bias).

#### Other outcomes

None of the other pre-specified outcomes were reported. However, one cohort study [39] reported that the change in blood glucose concentration was not different in infants fed EBM compared to infants who had other interventions (227 infants, MD [95% CI]: -1.4 [-3.7, 0.9]mg/dL, *p* = 0.25, good quality study). In addition, one RCT [35] reported that fasting blood glucose concentrations were lower at 24 h but higher at one to four weeks (measured weekly) after birth in infants fed EBM compared to infants fed formula (20 infants, high risk of bias, MD [95% CI] -0.52 [-0.77, -0.27] mmol/L, *p* < 0.0001 at 24 h; 1.02 [0.72, 1.32] mmol/L, *p* < 0.00001 at one week; 0.73 [0.49, 0.97] mmol/L, *p* < 0.00001 at two weeks; 1.14 [0.88, 1.40] mmol/L, *p* < 0.00001 at three weeks; 0.63 [0.36, 0.90] mmol/L, *p* < 0.00001 at four weeks).

### Outcomes for studies on the expression of breast milk

#### Neonatal hypoglycemia

**Fig. 3:**
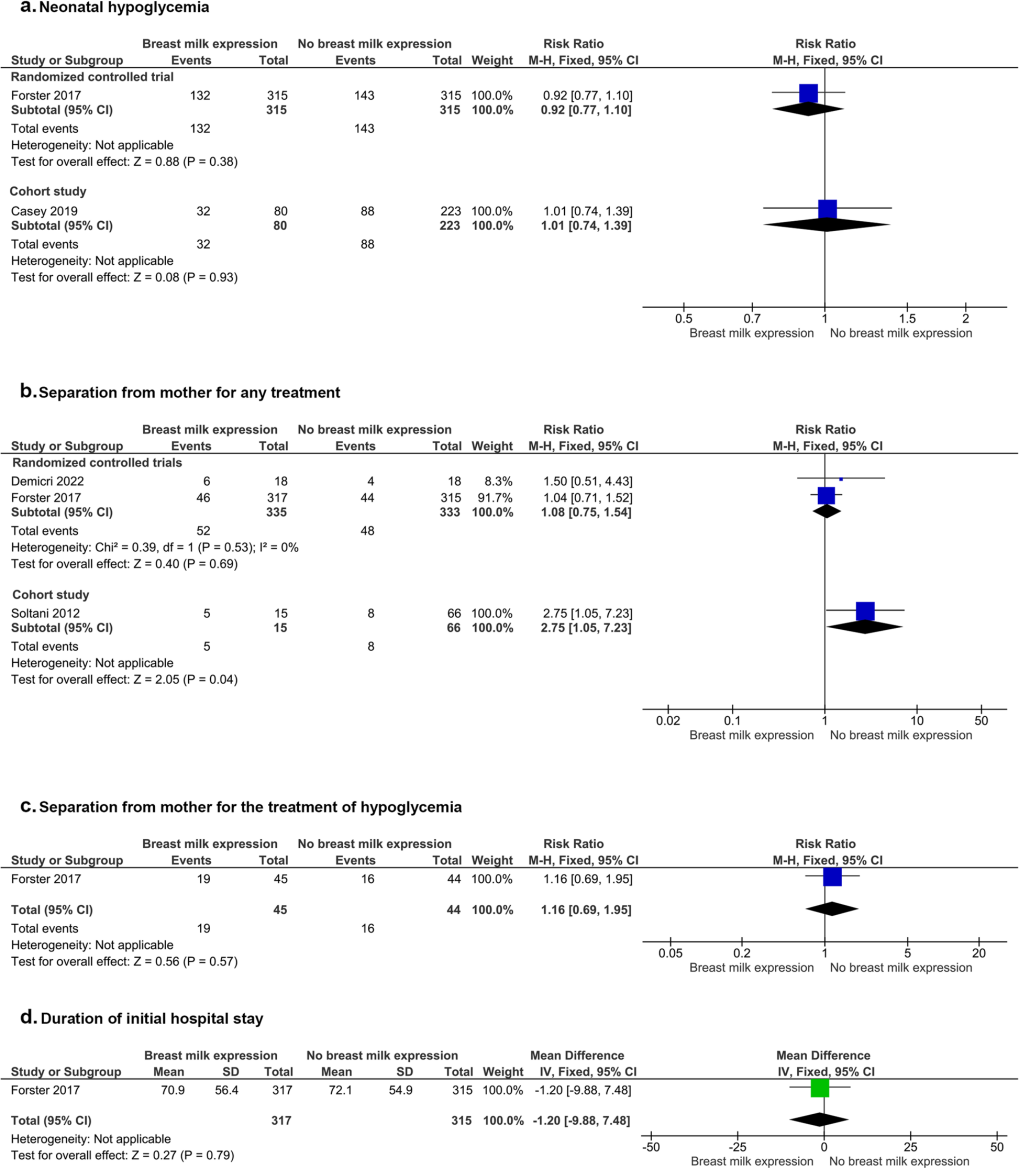
Breast milk expression and relationship with neonatal hypoglycemia, separation of infant from mother, and duration of hospital stay

Two studies (one RCT [16] and one cohort [40]) reported that the risk of hypoglycemia was not different in infants whose mothers expressed breast milk compared to infants whose mothers did not (RCT- 630 infants, RR [95% CI]: 0.92 [0.77, 1.10], *p* = 0.38, high certainty evidence, low risk of bias; cohort—303 infants, RR [95% CI]: 1.01 [0.74, 1.39], *p* = 0.93, poor quality study) (Fig. 3).

#### Separation from mother for any treatment

Two RCTs [16, 36] reported that the risk of separation from the mother for any treatment was not different between infants whose mothers expressed breast milk compared to infants whose mothers did not (2 studies, 668 infants, RR [95% CI]: 1.08 [0.75, 1.54], *p* = 0.69, I^2^ = 0; *P* = 0.53, low certainty evidence, 1 RCT at low risk of bias, the other at high risk of bias). In contrast, one cohort study [41] reported that infants whose mothers expressed breast milk had a higher risk of being separated from their mother (SCBU admission) compared to infants whose mothers did not express breast milk (81 infants, RR [95% CI]: 2.75 [1.05, 7.23], *p* = 0.04, poor quality study) (Fig. 3).

#### Separation from mother for the treatment of hypoglycemia

One RCT [16] reported the risk of separation from the mother for the treatment of hypoglycemia was similar among infants whose mothers did compared to infants whose mothers did not express breast milk antenatally (89 infants, RR [95% CI]: 1.16 [0.69, 1.95], *p* = 0.57, low certainty evidence, low risk of bias) (Fig. 3).

#### Duration of initial hospital stay

One RCT [16] reported no difference in the duration of initial hospital stay among infants whose mothers expressed breast milk antenatally compared to infants whose mothers did not (632 infants, MD [95% CI]: -1.20 [-9.88, 7.48] days, *p* = 0.79, low risk of bias) (Fig. 3).

#### Breastfeeding outcomes

**Fig. 4:**
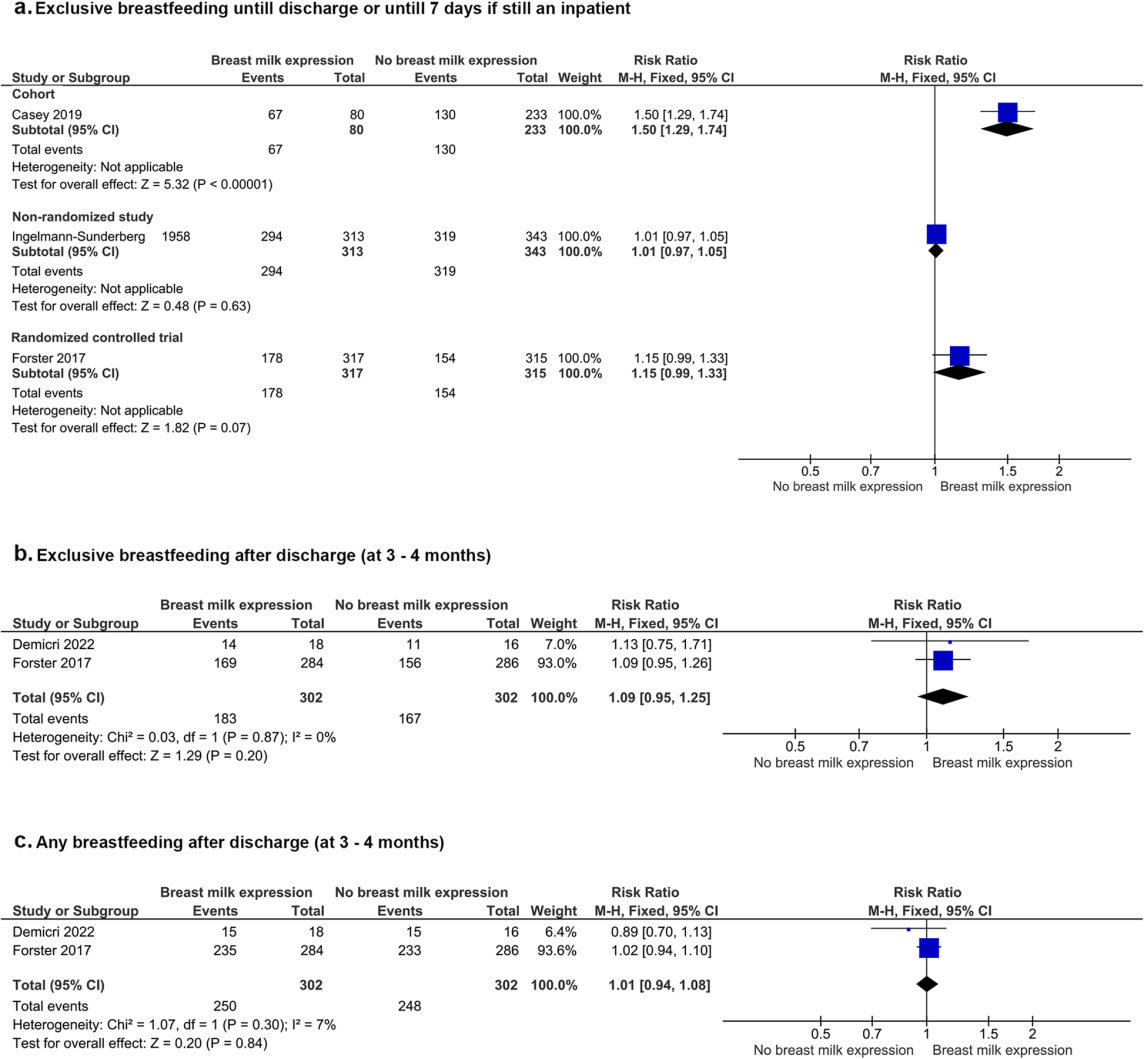
Breast milk expression and breastfeeding outcomes

Two studies (one RCT [16] and one NRSI [38]) reported that infants of mothers who expressed breast milk compared to infants whose mothers who did not were not more likely to be exclusively breast fed at discharge [38] or until seven days if still an in-patient [16] (RCT—632 infants, RR [95% CI]: 1.15 [0.99, 1.33], *p* = 0.07), some concerns about risk of bias; NRSI—656 infants, RR [95% CI]: 1.01 [0.97, 1.05], *p* = 0.63, serious risk of bias) (Fig. 4). In contrast, a cohort study [40] reported that infants whose mothers expressed breast milk compared to infants whose mothers did not were more likely to be exclusively breastfed until discharge (313 infants, RR [95% CI]: 1.50 [1.29, 1.74], *p* < 0.00001, poor quality study).

Two RCTs [16, 36] reported no significant difference in exclusive breast milk feeding rates at three to four months among infants whose mothers expressed breast milk antenatally compared to infants whose mothers did not (604 infants, RR [95% CI]: 1.09 [0.95, 1.25], *p* = 0.20, I^2^ = 0%; *P* = 0.87, some concerns about risk of bias with both studies) (Fig. 4).

Two RCTs [16, 36] reported that the rates of any breastfeeding three to four months after birth were similar in infants whose mothers expressed breast milk antenatally compared to infants whose mothers did not (604 infants, RR [95% CI]: 1.01 [0.94, 1.08], *p* = 0.30, I^2^ = 7%; *P* = 0.84, some concerns about risk of bias with both studies) (Fig. 4).

#### Other outcomes

None of our other pre-specified outcomes were reported by any of the five studies on breast milk expression.

The certainty of each GRADE outcome was assessed as very low, low, or high [29] (Table 4).

**Table 4:** GRADE outcomes for studies on expressed breast milk and the expression of breast milk. Patient or population: Infants (≤ 28 days old); Setting: Hospitals; Intervention: Expressed breast milk or expression of breast milk; Comparison: other or no intervention

Outcomes	Anticipated absolute effects^*^ (95% CI)		Relative effect (95% CI)	№ of participants (studies)	Certainty of the evidence (GRADE)	Comments
	**Risk with other or no intervention**	**Risk with Expressed breast milk**				
Expressed breast milk						
Neonatal hypoglycaemia	0 per 1 000	**0 per 1 000** (0 to 0)	not estimable	20 (1 RCT)	⨁◯◯◯ Very low^a,b^	For neonatal hypoglycemia, the benefit or harm of giving infants EBM could not be estimated as there were no hypoglycemic episodes in either group
Expression of breast milk						
Neonatal hypoglycaemia	454 per 1 000	**418 per 1 000** (350 to 499)	**RR 0.92** (0.77 to 1.10)	630 (1 RCT)	⨁⨁⨁⨁ High	Antenatal expression of breast milk results in little to no difference in neonatal hypoglycemia
Separation from mother for any treatment	144 per 1 000	**156 per 1 000** (108 to 222)	**RR 1.08** (0.75 to 1.54)	668 (2 RCTs)	⨁⨁◯◯ Low^c,d^	For separation of infant from mother for any treatment, benefit or harm with expression of breast milk could not be excluded
Separation from mother for treatment of hypoglycaemia	364 per 1 000	**422 per 1 000** (251 to 709)	**RR 1.16** (0.69 to 1.95)	89 (1 RCT)	⨁⨁◯◯ Low^d,e^	For separation of infant from mother for any treatment, benefit or harm with expression of breast milk could not be excluded

^*^The risk in the intervention group (and its 95% confidence interval) is based on the assumed risk in the comparison group and the **relative effect** of the intervention (and its 95% CI). **CI:** confidence interval

**GRADE Working Group grades of evidence: High certainty:** we are very confident that the true effect lies close to that of the estimate of the effect; **Moderate certainty:** we are moderately confident in the effect estimate: the true effect is likely to be close to the estimate of the effect, but there is a possibility that it is substantially different, **Low certainty:** our confidence in the effect estimate is limited: the true effect may be substantially different from the estimate of the effect, **Very low certainty:** we have very little confidence in the effect estimate: the true effect is likely to be substantially different from the estimate of effect

**Explanations:** ^a^Downgraded one level due to risk of bias as a result of no details on the randomization process

^b^Downgraded two levels due to imprecision as a result of the small sample size to detect differences and no event occurring in either group

^c^Downgraded one level due to risk of bias as one of the included studies is at high risk of bias because of missing outcome data and its measurement

^d^Downgrade one level due to imprecision as a result of the small sample size to detect differences

^e^Downgraded one level due to risk of bias as some concerns in the domain of deviations from the intended interventions

## Discussion

Our study has systematically reviewed the evidence for the effectiveness of giving EBM to infants and the mother’s expression of breast milk for prevention and treatment of neonatal hypoglycemia and other outcomes, including the duration of initial hospital stay, separation from the mother for any treatment or the treatment of hypoglycemia, and breastfeeding.

Despite the widespread practice and recommendations of feeding EBM to infants [5, 10, 12–14] and encouraging mothers to express breast milk [12, 14, 16] to prevent and treat neonatal hypoglycemia, we found no published study specifically designed to assess the effectiveness of these practices. However, a parallel group RCT is underway to determine the effectiveness of donor human milk supplementation in treating hypoglycemia in breastfed infants [30].

Breast milk, in addition to having adequate nutrients for optimal growth and development in the first six months of life, also has anti-infective, immunomodulatory, and anti-inflammatory benefits  [42, 44], which are associated with improved short- and long-term health outcomes [43]. However, there have been conflicting reports on whether it increases blood glucose concentrations. Rees et al. [44] reported that among breastfed infants, there was a significant increase in blood glucose concentrations of 9.6 mg/dL when fed donor human milk (DHM) and 7.8 mg/dL when fed formula. In contrast, Harris et al. [39] reported a significant increase in blood glucose concentration following formula feeds but no change in the blood glucose concentration of hypoglycemic infants fed mother’s EBM in the first 48 h after birth. This could be because of the different sources of breast milk. There have been concerns about the adequacy of volume and hence available calories of mother’s milk in the first few days after birth, since lactation is often not well established in this period [45]. For example, Harris et al. [39] reported that the median breast milk volume (0.5 mL/kg) available to feed infants was substantially smaller than the median volume of formula (4.5 mL/kg) given to the infants. Since the greatest risk of neonatal hypoglycemia is in the first few days after birth, when maternal lactation may not be well established, to determine the effectiveness of EBM in preventing and treating neonatal hypoglycemia, future studies should consider the use of donor human milk as a supplement to mother’s milk, if required.

Our finding that breast milk expression had no significant effect on neonatal hypoglycemia may be surprising because the expression of breast milk provides milk feeds for the infant and also potentially improves the initiation and establishment of lactogenesis [46]. The authors of the RCT [16] that reported this outcome, however, noted that while the volume of milk expressed by mothers ranged from zero to 905mls, the number of expressing episodes ranged from one to 59 times. The wide variation in these variables may be responsible for the reported lack of benefit on neonatal hypoglycemia. Further studies are needed to determine the optimal frequency of expression and breast milk volume required to potentially prevent and treat neonatal hypoglycemia.

While the RCTs [16, 36] included in our review showed with low certainty that maternal expression of breast milk was not significantly associated with the separation of the infant from the mother for any treatment or for the treatment of hypoglycemia, the included cohort study [41] reported that infants whose mothers expressed breast milk had more than a two-and-a-half times higher risk of being separated from their mothers. The authors of this cohort study attributed this to the lower gestational age at birth in the group of infants whose mothers expressed breast milk. While it has been hypothesized that the expression of breast milk causes the release of oxytocin, which may lead to preterm birth, several other studies have not shown a significant reduction in the gestational age at birth of infants whose mothers expressed breast milk antenatally compared to infants who did not [16, 36, 40].

The NRSI [38] at high risk of bias showed a benefit of antenatal expression of breast milk on exclusive breastfeeding at discharge, and the RCT by Forster et al. [16] reported that antenatal expression of breast milk is effective in achieving exclusive breast milk feeding in the first 24 h after birth. However, other included studies (RCTs) showed neither benefit nor harm of antenatal breast milk expression on exclusive breastfeeding until discharge, or three-to-four months, or any breastfeeding at three-to-four months, suggesting that any possible short-term benefits of antenatal expression of breast milk on exclusive breastfeeding do not persist after the first few days.

Our systematic review and meta-analysis has some strengths. To the best of our knowledge, this is the first systematic review to determine the effectiveness of EBM and breast milk expression in preventing and treating neonatal hypoglycemia, although these are widely recommended and practised. As mechanisms that produce the desired outcomes may differ for these two interventions which are often linked, it is essential that studies on EBM and its expression are considered separately, as these are both recommended in many neonatal hypoglycemia management guidelines. Similarly, we have identified important knowledge and logistic gaps to be considered in future studies that may be designed to determine the effectiveness of EBM and the expression of breast milk in preventing and treating neonatal hypoglycemia.

Our study also has limitations. Although some studies with variable risk of bias in our review reported the prevalence of hypoglycemia, none were specifically designed to determine the effectiveness of the interventions for preventing and treating neonatal hypoglycemia. This underscores the need for more focused, high-quality studies. Similarly, all studies we reviewed on breastfeeding outcomes either had some concerns or were at high risk of bias for these outcomes, and our findings on breastfeeding outcomes need to be interpreted with this in mind. Thirdly, many outcomes of interest (number and severity of hypoglycemic episodes, injury attributable to hypoglycemia on neuroimaging, cost of intervention, and cost of neonatal care) were not reported in the included studies. Hence, we could not synthesize any evidence on these outcomes.

## Conclusions

Given the few studies with variable risk of bias, we found insufficient evidence for the effectiveness of EBM for the prevention and treatment of neonatal hypoglycemia. There is high certainty evidence that breast milk expression may not alter the risk of neonatal hypoglycemia, and low certainty evidence of no benefit nor harm for the separation of the infant from the mother for any treatment or the treatment of hypoglycemia. Further high-quality RCTs are needed that are specifically designed to determine the effectiveness of EBM and breast milk expression in preventing and treating neonatal hypoglycemia and report on other important outcomes, including number and severity of hypoglycemic episodes, injury attributable to hypoglycemia on neuroimaging, cost of intervention, and cost of neonatal care.

### Supplementary Information

**Additional file 1.** Search strategy for databases and Clinical Trial Registries. Data contains the search strategy (key words and MESH terms) used in this systematic review.

## Data Availability

Data access requests are to be submitted to the Data Access Committee via researchhub@auckland.ac.nz. Data will be shared with researchers with a sound proposal on reasonable request.

## Abbreviations

EBM: Expressed breast milk
RCT: Randomised controlled trial
NRSI: Non-randomised study of intervention
PROSPERO: International prospective register of systematic reviews
PRISMA: Preferred Reporting Items for Systematic Reviews and Meta‐Analyses
ROBINS-I: Risk Of Bias In Non-randomized Studies of Interventions tool
NICU: Neonatal Intensive Care Unit
SCBU: Special care baby unit
I^2^: Inconsistency Index
GRADE: Grading of Recommendations Assessment, Development and Evaluation
DHM: Donor human milk
