# Supplementary material for: Expressed breast milk and maternal expression of breast milk for the prevention and treatment of neonatal hypoglycemia: a systematic review and meta-analysis
Source: Matern Health Neonatol Perinatol. 2023 Oct 9;9:12. doi: 10.1186/s40748-023-00166-0 (PMC10561482; doi:10.1186/s40748-023-00166-0)
Supplement: Supplementary file 1 — Additional file 1. Search strategy for databases and Clinical Trial Registries. Data contains the search strategy (key words and MESH terms) used in this systematic review. [file 40748_2023_166_MOESM1_ESM.docx]

**Databases**

Ovid MEDLINE(R) Epub Ahead of Print, In Process & Other Non-Indexed Citations, Ovid MEDLINE (R) Daily, and Ovid MEDLINE (R) 1946-Present

| **#** | **Query** |
| --- | --- |
| 1 | exp Infant/ |
| 2 | (baby* or babies or infant or infants or infant? or infantile or infancy or low birth weight or low birthweight or neonat* or newborn* or new born or new borns or newly born or premature or prematures or prematurity or preterm or preterms or pre term or preemie or preemies or premies or premie or VLBW or LBW or ELBW).mp. [mp=title, abstract, original title, name of substance word, subject heading word, floating sub-heading word, keyword heading word, organism supplementary concept word, protocol supplementary concept word, rare disease supplementary concept word, unique identifier, synonyms] |
| 3 | 1 or 2 |
| 4 | Milk, Human/ or Breast Milk Expression/ |
| 5 | Milk Banks/ |
| 6 | (((donor* or donat* or shar* or bank*) adj3 (human milk or breast?milk)) or DHM or DBM or human milk).mp. [mp=title, abstract, original title, name of substance word, subject heading word, floating sub-heading word, keyword heading word, organism supplementary concept word, protocol supplementary concept word, rare disease supplementary concept word, unique identifier, synonyms] |
| 7 | (expres* breast milk or breast milk expres* or human milk or donor milk).mp. [mp=title, abstract, original title, name of substance word, subject heading word, floating sub-heading word, keyword heading word, organism supplementary concept word, protocol supplementary concept word, rare disease supplementary concept word, unique identifier, synonyms] |
| 8 | 4 or 5 or 6 or 7 |
| 9 | Hypoglycemia/ |
| 10 | hypoglyc*.mp. [mp=title, abstract, original title, name of substance word, subject heading word, floating sub-heading word, keyword heading word, organism supplementary concept word, protocol supplementary concept word, rare disease supplementary concept word, unique identifier, synonyms] |
| 11 | (low glucose or low blood sugar or low blood glucose).mp. [mp=title, abstract, original title, name of substance word, subject heading word, floating sub-heading word, keyword heading word, organism supplementary concept word, protocol supplementary concept word, rare disease supplementary concept word, unique identifier, synonyms] |
| 12 | Blood Glucose/ |
| 13 | 9 or 10 or 11 or 12 |
| 14 | Intensive Care, Neonatal/ |
| 15 | (neonatal intensive care or neonatal intensive care unit or nicu or special care baby unit or scbu or neonatal nursery or lying-in ward or rooming in).mp. [mp=title, abstract, original title, name of substance word, subject heading word, floating sub-heading word, keyword heading word, organism supplementary concept word, protocol supplementary concept word, rare disease supplementary concept word, unique identifier, synonyms] |
| 16 | 14 or 15 |
| 17 | 13 or 16 |
| 18 | 3 and 8 and 17 |

2)Embase

Embase <1980 to 2022 May 19>

| **#** | **Query** |
| --- | --- |
| 1 | exp infant/ |
| 2 | (baby* or babies or infant or infants or infant? or infantile or infancy or low birth weight or low birthweight or neonat* or newborn* or new born or new borns or newly born or premature or prematures or prematurity or preterm or preterms or pre term or preemie or preemies or premies or premie or VLBW or LBW or ELBW).mp. [mp=title, abstract, heading word, drug trade name, original title, device manufacturer, drug manufacturer, device trade name, keyword heading word, floating subheading word, candidate term word] |
| 3 | 1 or 2 |
| 4 | breast milk/ or breast milk expression/ |
| 5 | milk bank/ |
| 6 | (((donor* or donat* or shar* or bank*) adj3 (human milk or breast?milk)) or DHM or DBM or human milk).mp. [mp=title, abstract, heading word, drug trade name, original title, device manufacturer, drug manufacturer, device trade name, keyword heading word, floating subheading word, candidate term word] |
| 7 | (expres* breast milk or breast milk expres* or human milk or donor milk).mp. [mp=title, abstract, heading word, drug trade name, original title, device manufacturer, drug manufacturer, device trade name, keyword heading word, floating subheading word, candidate term word] |
| 8 | 4 or 5 or 6 or 7 |
| 9 | hypoglycemia/ |
| 10 | hypoglyc*.mp. [mp=title, abstract, heading word, drug trade name, original title, device manufacturer, drug manufacturer, device trade name, keyword heading word, floating subheading word, candidate term word] |
| 11 | (low glucose or low blood sugar or low blood glucose).mp. [mp=title, abstract, heading word, drug trade name, original title, device manufacturer, drug manufacturer, device trade name, keyword heading word, floating subheading word, candidate term word] |
| 12 | blood glucose.mp. or glucose blood level/ |
| 13 | 9 or 10 or 11 or 12 |
| 14 | newborn intensive care/ |
| 15 | (neonatal intensive care or neonatal intensive care unit or nicu or special care baby unit or scbu or neonatal nursery or lying-in ward or rooming in).mp. [mp=title, abstract, heading word, drug trade name, original title, device manufacturer, drug manufacturer, device trade name, keyword heading word, floating subheading word, candidate term word] |
| 16 | 14 or 15 |
| 17 | 13 or 16 |
| 18 | 3 and 8 and 17 |

3)Cinahl plus_19_5_22 (history 1961 to present; temporal coverage 1937 to present)

(MH "Infant+") OR (baby* or babies or infant or infants or infant? or infantile or infancy or low birth weight or low birthweight or neonat* or newborn* or newborn or new borns or newly born or premature or prematures or prematurity or preterm or preterms or pre term or preemie or preemies or premies or premie or VLBW or LBW or ELBW)

(MM "Milk Expression") OR (MM "Milk, Human") OR (MM "Milk Banks") OR (((donor* or donat* or shar* or bank*) N3 (human milk or breast?milk)) or DHM or DBM or human milk) OR (expres* breast milk or breast milk expres* or human milk or donor milk)

((MM "Hypoglycemia") OR hypoglyc* OR (MM "Blood Glucose") OR (low glucose or low blood sugar or low blood glucose)) OR (((MM "Intensive Care, Neonatal")) or (neonatal intensive care or neonatal intensive care unit or nicu or special care baby unit or scbu or neonatal nursery or lying-in ward or roomimg in))

4)Scopus (51.3 million records post-1995 with references, 25.3 million records pre-1996, with the oldest record dating back to 1788)

baby* or babies or infant or infants or infant? or infantile or infancy or “low birth weight” or “low birthweight” or neonat* or newborn* or “new born” or “new borns” or “newly born” or premature or prematures or prematurity or preterm or preterms or “pre term” or preemie or preemies or premies or premie or VLBW or LBW or ELBW

AND

(((donor* or donat* or shar* or bank*) W/3 (“human milk” or breast?milk)) or DHM or DBM or “human milk”) or (“expres* breast milk” or “breast milk expres*” or “human milk” or “donor milk”)

AND

hypoglyc* OR “low glucose” or “low blood sugar” or “low blood glucose” OR “blood glucose” OR “neonatal intensive care” or “neonatal intensive care unit” or nicu or “special care baby unit” or scbu or “neonatal nursery” or “lying-in ward” or “rooming in”

( TITLE-ABS-KEY ( baby*  OR  babies  OR  infant  OR  infants  OR  infant?  OR  infantile  OR  infancy  OR  "low birth weight"  OR  "low birthweight"  OR  neonat*  OR  newborn*  OR  "new born"  OR  "new borns"  OR  "newly born"  OR  premature  OR  prematures  OR  prematurity  OR  preterm  OR  preterms  OR  "pre term"  OR  preemie  OR  preemies  OR  premies  OR  premie  OR  vlbw  OR  lbw  OR  elbw )  AND  TITLE-ABS-KEY ( ( ( ( donor*  OR  donat*  OR  shar*  OR  bank* )  W/3  ( "human milk"  OR  breast?milk ) )  OR  dhm  OR  dbm  OR  "human milk" )  OR  ( "expres* breast milk"  OR  "breast milk expres*"  OR  "human milk"  OR  "donor milk" ) )  AND  TITLE-ABS-KEY ( hypoglyc*  OR  "low glucose"  OR  "low blood sugar"  OR  "low blood glucose"  OR  "blood glucose"  OR  "neonatal intensive care"  OR  "neonatal intensive care unit"  OR  nicu  OR  "special care baby unit"  OR  scbu  OR  "neonatal nursery"  OR  "lying-in ward"  OR  "rooming in" ) )

5)Cochrane Library

baby* or babies or infant or infants or infant? or infantile or infancy or “low birth weight” or “low birthweight” or neonat* or newborn* or “new born” or “new borns” or “newly born” or premature or prematures or prematurity or preterm or preterms or pre term or preemie or preemies or premies or premie or VLBW or LBW or ELBW

AND

(((donor* or donat* or shar* or bank*) NEAR/3 (“human milk” or breast?milk)) or DHM or DBM or “human milk”) or (“expres* breast milk” or “breast milk expres*” or “human milk” or “donor milk”)

AND

hypoglyc* OR “low glucose” or “low blood sugar” or “low blood glucose” OR “blood glucose” OR “neonatal intensive care” or “neonatal intensive care unit” or nicu or “special care baby unit” or scbu or “neonatal nursery” or “lying-in ward” or “rooming in”

Trial websites

Current Controlled Trials, Clinical Trials, Australian and New Zealand Clinical Trials Registry, WHO ICTRP Search Portal

Search words: Expressed breast milk, expressed breastmilk, breast milk expression, breastmilk expression
